# Anthelmintic Effects of Alkylated Diamines and Amino Alcohols against *Schistosoma mansoni*

**DOI:** 10.1155/2013/783490

**Published:** 2013-08-20

**Authors:** Fábio de Souza Fernandes, Celso O. Rezende Júnior, Tayrine Silva Fernandes, Lígia Souza da Silveira, Carlos A. M. Rezende, Mauro V. De Almeida, Renato G. de Paula, Vanderlei Rodrigues, Ademar A. Da Silva Filho, Mara R. C. Couri

**Affiliations:** ^1^Departamento de Química, Universidade Federal de Juiz de Fora, 36036-330 Juiz de Fora, MG, Brazil; ^2^Departamento de Bioquímica e Imunologia, Universidade de São Paulo, 14049-900 Ribeirão Preto, SP, Brazil; ^3^Departamento de Ciências Farmacêuticas, Faculdade de Farmácia, Universidade Federal de Juiz de Fora, 36036-900 Juiz de Fora, MG, Brazil

## Abstract

Polyamines are substances involved in many aspects of cell growth, division, and differentiation. Because of the metabolic differences between host cells and parasite cells, polyamine metabolism has been considered as a potential target for the chemotherapy of parasitic diseases. The aim of this work was to evaluate the schistosomicidal activity of different *N*-alkylated diamines (**3a**–**3h**), amino alcohols (**4a**–**4d**), and glycosylated amino alcohols (**10a**–**10d**). Compounds were prepared by synthetic methods and submitted to *in vitro* evaluation against adult worms of *Schistosoma mansoni*. At 100 *μ*M, **3b**, **3e**, and **3h** as well as **4a**, **4b**, **4d**, **10a**, **10b**, and **10d** resulted in 100% mortality of adult schistosomes. Compound **3d** (12.5 to 100 *μ*M) caused the death of 100% of both male and female adult schistosomes, while **3f** (12.5 to 100 *μ*M) resulted in 100% mortality of only male adult worms, whereas no mortality in female worms was observed. Compounds **3d** and **3f** were also able to reduce viability and decrease production of developed eggs in comparison with the negative control group. Diamines **3d** and **3f** may represent useful lead compounds for further optimization in order to develop new schistosomicidal agents.

## 1. Introduction

Human schistosomiasis is a chronic liver and intestinal parasitic disease caused by blood flukes of the genus *Schistosoma*, mainly *S. mansoni* [1]. Schistosomiasis, endemic in approximately 77 countries, is considered to be one of the most significant neglected tropical diseases in the world, affecting more than 200 million people worldwide [2]. The only drug used in treatment of schistosomiasis is praziquantel (PZQ), which does not prevent reinfection and is inactive against juvenile schistosomes [2, 3]. In addition, there is a considerable concern about the development of praziquantel resistance [3, 4]. This scenario emphasizes the increasing need for the development of novel and inexpensive drugs against schistosomiasis. In order to provide new hit/lead structures, which can be used in drug development to control schistosomiasis, the search for schistosomicidal compounds, mainly from natural sources, has been intensified in the last years [5–8]. 

Polyamines are substances that occur widely in biological material and are thought to be involved in many aspects of cell, such as growth, division, and differentiation [9]. Because of the metabolic differences between host cells and parasite cells, polyamine metabolism has been considered as a potential target for the chemotherapy of parasitic diseases [10].

Regarding the activity of amine compounds, several authors have shown the antiparasitic activity of different *N*-alkylated diamines and amino alcohols [10–17]. In a previous study, it was shown that lipophilic diamine and amino alcohol derivatives display inhibitory effects on promastigote forms of *Leishmania chagasi* and *Leishmania amazonensis* [12, 16]. It was also reported that this series of compounds showed trypanocidal activity against trypomastigotes forms of *Trypanosoma cruzi*, showing that compounds containing alkyl chains with 12 carbon atoms displayed similar activity to the reference drug crystal violet [17]. Moreover, Penido et al. reported the *in vitro* and *in vivo* schistosomicidal activity of a series of alkylaminothiosulfuric compounds [18].

Considering the design of novel antiparasitic drugs based on polyamine compounds and amino alcohol derivatives, lipophilicity is an important parameter. The introduction of long alkyl chains may enhance the ability of compounds in interacting with membrane lipids, allowing their penetration into the parasite and, consequently, modifying either the polyamine transport or the metabolism of the parasite. Also, the insertion of carbohydrates into amino alcohol molecules may be useful for disturbing cell integrity, since glycosylated derivatives could be considered nonionic surfactants compounds [9–17, 19].

In this context, in continuation of our search for bioactive schistosomicidal compounds [20, 21] and on the basis of their antiparasitic potential, this work describes the *in vitro* schistosomicidal activity of a series of lipophilic diamines and amino alcohols, as well as glycosylated amino alcohols derivatives against adult worms of* Schistosoma mansoni*.

## 2. Materials and Methods

### 2.1. General Methods

IR spectra were recorded using a BOMEM-FTIR MB102 spectrometer (AABB Bomem Inc., QC, Canada). ^1^H and ^13^C NMR spectra were recorded on Bruker Advance DRX300 spectrometer (Bruker Corporation, Billerica, MA, USA). Thin-layer chromatography (TLC) was performed on glass plates and silica gel sheets (Silica Gel F254; Merck, Whitehouse Station, NK, USA), visualized with iodine vapor, and/or revealed with ethanolic H_2_SO_4_ solution. Column chromatography was carried out on silica gel 60 (E. Merck 70–230 mesh). Solvents were purchased from Vetec Química (Vetec, Xerem, RJ, Brazil) and were distilled prior to use. Reagents were purchased from Aldrich (Sigma Aldrich, Saint Louis, MI, USA) and used without further purification. Log *P* was calculated using Chemdraw Ultra 12 software (trial version).

### 2.2. Synthesis of Alkylated Diamines and Amino Alcohols

**Scheme 1:**
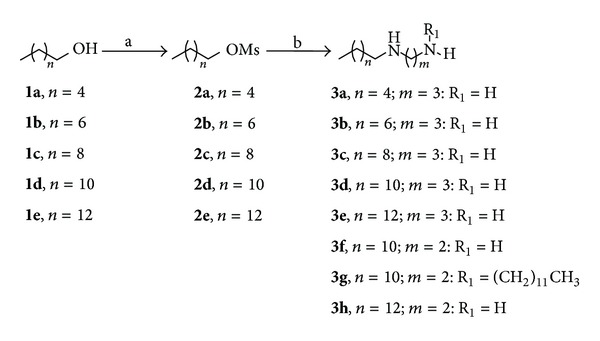
Synthesis of *N*-alkylated and *N*,*N*′-dialkylated diamines: (a) MsCl, CH_2_Cl_2_, Py, 0°C to room temperature; (b) diamine, EtOH reflux.

**Scheme 2:**
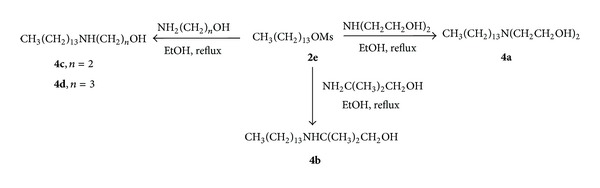
Synthesis of* N*-alkylated amino alcohols.

**Scheme 3:**
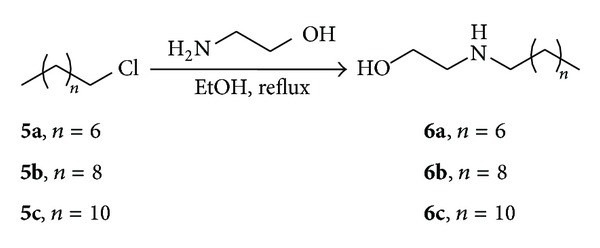
Synthesis of *N*-alkylated amino alcohols.

*N*-mono, *N*,*N*′-dialkylated diamines (Scheme 1) and *N*-monoalkylated amino alcohols (Scheme 2) were prepared using a similar methodology previously described [10–14]. For the preparation of the *N*-mono and *N*,*N*′-dialkylated diamines (Scheme 1), alcohols **1a**–**e** were first mesylated in pyridine, leading to compounds **2a**–**e**. These mesylated derivatives were then treated with 1,2-ethanediamine or 1,3-propanediamine in ethanol under reflux, furnishing the desired compounds **3a**–**3h**. For the preparation of *N*-monoalkylated amino alcohols **4a**–**d** (Scheme 2), the mesylated derivative **2e** was treated with commercial amino alcohols (diethanolamine, 2-amine-2-methylpropan-1-ol, ethanolamine, and 3-aminopropan-1-ol). For the obtainment of *N*-alkylated compounds **6a**–**c **(Scheme 3) alkyl chlorides **5a**–**c** were treated with ethanolamine in ethanol at reflux for 24 h furnishing the desired compounds. The alkylated diamines and amino alcohols were purified by column chromatography or recrystallization. The structures were assigned by ^1^H NMR and ^13^C NMR experiments [10–14].

### 2.3. Synthesis of Glycosylated Derivatives

**Scheme 4:**
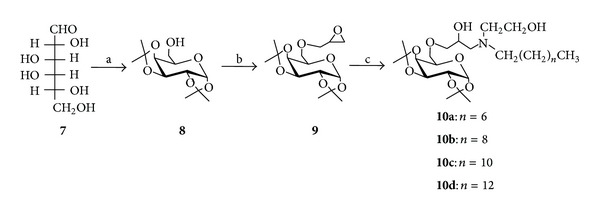
Preparation of D-galactose derivatives. Reagents and conditions: (a) acetone, H_2_SO_4_, ZnCl_2_ (58%); (b) NaOH 40%, TBAB, Epichlorohydrin, THF (94%); (c) CH_3_(CH_2_)_*n*_CH_2_NHCH_2_CH_2_OH, EtOH, TBAB, rt (51–61%).

D-Galactose **7** was converted into 6-*O*-[2,3-epoxypropyl]-1,2 : 3,4-di-*O*-isopropylidene-*α*-D-galactopyranose **9** according to the literature procedure [22, 23] and then treated with amino alcohols **4c** (Scheme 2) e **6a**–**c** (Scheme 3) in presence of tetrabutyl ammonium bromide (TBAB) in EtOH at room temperature (Scheme 4). No attempt was made to determine the stereochemistry of compounds **10a**–**d** as they were prepared from the corresponding racemic epichlorohydrin. All the compounds were purified by column chromatography. The structures were assigned by infrared and ^1^H and ^13^C NMR experiments [22, 23]. 

### 2.4. Parasite Culture and Maintenance

 The LE (Luiz Evangelista) strain of *S. mansoni *was maintained by passage through *Biomphalaria glabrata *snails and Balb/c mice. After eight weeks, *S. mansoni* adult worms (male and female) were recovered under aseptic conditions from mice previously infected with 200 cercariae by perfusion of the livers and mesenteric veins [20, 21]. The worms were washed in RPMI 1640 medium (Invitrogen), kept at pH 7.5 with HEPES 20 mM, and supplemented with penicillin (100 UI·mL^−1^), streptomycin (100 *μ*g·mL^−1^), and 10% bovine fetal serum (Gibco). After washing, one pair of adult worms was transferred to each well of a 24-well culture plate containing 2 mL of the same medium and incubated at 37°C in a humid atmosphere containing 5% CO_2_ prior to use. All experiments were authorized by the Ethical Committee for Animal Care of University of São Paulo (Approval no.: 021/2009, June 2, 2009) in accordance with the national and international accepted principles for laboratory animal use and care.

### 2.5. *In Vitro* Studies with *S. mansoni*

For the *in vitro *test with *S. mansoni*, a preliminary screening of compounds was performed at 100 *μ*M [20, 21]. The active samples were further studied at lower concentrations. Samples were dissolved in DMSO and used at concentrations ranging from 12.5 to 100 *μ*M (compounds). Solutions of samples were added to the RPMI 1640 medium containing one adult worm pair after a period of 24 h of adaptation to the culture medium. The parasites were kept for 72 h and monitored every 24 h in order to evaluate their general condition (motor activity and mortality rate), egg production, and egg development. Significant alteration in motor activity was defined as minimal movement observed for 1 minute [5–8]. The worms were considered dead when no movement was observed for at least 2 minutes of examination [20, 21]. Changes in the pairing were also evaluated using an inverted microscope (Leitz) [5–8, 20, 21]. All experiments were carried out in quadruplicate and repeated at least four times, using 10 *μ*M praziquantel (PZQ) as positive control group and RPMI 1640 medium and RPMI 1640 with 0.4% DMSO as negative control groups.

### 2.6. Viability Assay

Pairs of adult worms were incubated for 72 h with active compounds (12.5, 25, 50, and 100 *μ*M), and their viability was assessed using the MTT assay [20, 21]. After incubation, each pair of adult worms was placed individually into wells (96-well plates) containing 100 *μ*L of phosphate-buffered saline (PBS) with 5 mg of MTT per milliliter for 30 min at 37°C. The solution was carefully removed and replaced with 200 *μ*L of DMSO, and the worms were allowed to stand in DMSO at room temperature for 1 h. The absorbance was read at 550 nm using as negative control groups RPMI 1640 medium and RPMI 1640 with 0.4% DMSO. PZQ (10 *μ*M) was used as positive control group. The percentage of viability was calculated in relation to the negative control group. 

### 2.7. Statistical Analysis

Results were expressed as mean ± S.E.M. Data were statistically analyzed by one-way analysis of variance, followed by Dunnett's test, with the level of significance set at *P* < 0.05.

## 3. Results and Discussion

Alkylated diamines and amino alcohols have been reported to possess a wide range of biological activities that include antibacterial, leishmanicidal, and trypanocidal [10–17]. However, to our knowledge, the schistosomicidal activity of alkylated diamines and amino alcohols is now being reported for the first time in this study. In this work, several diamines and amino alcohols compounds that had been reported to possess antiparasitic and/or antimicrobial activities were selected for our antischistosomal experiments.

The compounds **3a**–**3e**, derived from 1,3-propanediamine, with lipophilic chain from 4 to 12 carbon atoms, were synthesized to evaluate the influence of lipophilicity on activity (Scheme 1). To evaluate the influence of the spacing between nitrogen atoms on schistosomicidal activity, the compounds **3f**–**h**, derived from 1,2-ethanediamine, were synthesized (Scheme 1). The amino alcohols **4a**–**4d** with lipophilic chains of 14 carbon atoms were synthesized to evaluate the influence of amino alcohol groups on schistosomicidal activity (Scheme 2). On the other hand, glycosylated amino alcohols (**10a**–**10d**) (Scheme 4) were synthesized to evaluate the influence of the carbohydrate subunit in the schistosomicidal activity [22, 23].

In a preliminary survival of 56-day-old adult worms of *S. mansoni* test, alkylated diamines (**3a**–**3h**), amino alcohols (**4a**–**4d**), and glycosylated amino alcohols (**10a**–**10d**) were tested at 100 *μ*M. As can be observed in Table 1, diamines **3b**, **3d**, **3e**, **3f**, and **3 h** as well as the amino alcohols **4a**, **4b**, **4d**, **10a**, **10b**, and **10d** showed significant schistosomicidal activities when tested at 100 *μ*M, causing the death of 100% of *S. mansoni* adult worms. No significant results were found for alkylated diamines **3a**, **3c**, **3g**, and amino alcohols **4c** and **10c**, which were not able to kill all adult parasites. As shown in Table 1, PZQ (10 *μ*M) caused 100% mortality, whereas no effect was observed in worms in the control (RPMI 1640 medium) and vehicle (RPMI medium plus 0.4% DMSO) groups.

A correlation between biological activity and hydrophobic character is frequently observed. The partition coefficient *P* of a compound in an *n*-octanol/water system represents the hydrophobic properties of this compound and can be determined experimentally or calculated. Considering the number of dead worms for the compounds, at a concentration of 100 *μ*M, a correlation between number of dead worms (%) and log (*P*) may be established (Table 1). All active compounds, at 100 *μ*M, showed log (*P*) between 1.99 and 5.30, including compounds **3d** (log *P* of 3.65) and **3f** (log *P* of 3.65), excepting compounds **3c** and **4c**, which were inactive. Interestingly, the diamine **3c**, with intermediate lipophilic chain, as well as compounds **3a** (log *P* of 1.15) and **3g** (log *P* of 8.65), which have lower and higher lipophilic chains, respectively, were inactive.

The active compounds in preliminary assays were further tested at lower concentrations (ranging from 12.5 to 50 *μ*M), as presented in Table 2. Regarding mortality rate, when compound **3d** was tested at 12.5 to 50 *μ*M, it caused the death of 100% of both male and female *S. mansoni* adult worms, after 24 h of incubation. In contrast, the exposure to 12.5 to 50 *μ*M of compound **3f** resulted in 100% mortality of male adult worms, whereas no mortality in the female worms was observed. Similarly, glycosylated amino alcohols **10b** and **10d**, at 25 to 50 *μ*M, caused the death of 100% of male adult worms and 25% mortality of female schistosomes, but at 12.5 *μ*M, no impact on mortality of adult worms was observed after exposure to **10b** and **10d**. Additionally, **4a** and **10a** (50 *μ*M) were able to cause 75% mortality of male and 25% mortality of female adult worms. Moreover, no significant effects on mortality were found after incubation with compounds **3b**, **3e**, **3h**, **4b**, and **4d** at concentrations of 12.5 to 50 *μ*M (Table 2), and morphological analysis revealed no tegumentary changes after incubation of both male and female *S. mansoni* adult worms with any tested compounds (data not shown). 

It is important to emphasize that male worms were more susceptible than female worms to the active compounds **10b**, **10c**, and especially for **3f**. Interestingly, compound **3d**, which is chemically similar to **3f**, caused the death of 100% of adult schistosome with no distinction between male and female. A similar variation in drug susceptibility between male and female schistosomes has been observed with several antischistosomal drugs [4–8, 20, 21, 24]. Similarly, recent studies on the effects of the essential oil of *Ageratum conyzoides*, ginger extract (*Zinger officinale*), and PZQ on *S. mansoni* showed that male worms tended to be more susceptible than female worms [8, 24–27]. In contrast, results with artesunate showed higher survival rates for male than for female worms [28]. Remarkably, our data demonstrated that compound **3f** showed an optimal *in vitro* activity against adult stage of* S. mansoni*, exhibiting high differential sensitivity to male worms at all tested concentrations (12.5 to 50 *μ*M).

On the other hand, no impact on worm motor activity was observed in the groups treated with no lethal concentrations of all active compounds (Table 2). Also, schistosomes maintained in RPMI 1640 medium or in RPMI medium plus 0.4% DMSO kept a normal motor activity. In contrast, all parasites belonging to the positive control group (PZQ, 10 *μ*M) had a significant decrease in motor activity. In this regard, it has been reported that the motility of *S. mansoni* is associated with important neurotransmitters or neuromodulators such as serotonin, dopamine, acetylcholine, epinephrine, neuropeptides, and glutamate [29–31]. Thus, it is suggested that mortality rate of adult schistosomes exposed to these tested diamines and amino alcohols might not be associated with alterations in the neurotransmitter or neuromodulator system of the parasite.

In addition, considering human schistosomiasis, its pathology is not due directly to the adult worm but rather to the large numbers of eggs that become trapped in the host's tissues, during egg migration [25–29, 32]. Regarding this, the analysis of the effects of schistosomicidal compounds on the reproductive fitness of *S. mansoni *may be an important strategy used to discover new drugs [32, 33]. In this sense, the reproductive fitness of *S. mansoni *is assessed by (i) pairing, an indicator of the mating process; and (ii) egg production, an indicator of egg output per worm [32, 33]. According to the literature, the permanent pairing of the schistosomes couples in the blood system of their hosts vertebrates throughout their lifespan causes high rate of oviposition, which is responsible for the resulting immunopathological lesions, characterized by inflammation and fibrosis in the target organ [8, 34]. Furthermore, in order to evaluate the schistosomicidal effect of a drug, it is important to analyze several parameters, such as motility, oviposition, and mortality [25, 32]. Also, according to Moraes et al. (2011), compounds with schistosomicidal activity can be effective in different ways: prophylactically (causing the death of schistosomula), suppressively (inhibiting oviposition), and curatively (causing the death of the adult worm) [31–33].

Regarding changes in the pairing, all pairs of coupled adult worms were separated into individual male and female after incubation with 25 to 50 *μ*M of compounds **3e** and **4b**, while 50% of the pairs remained coupled after exposure of compounds **3d** (12.5 to 50 *μ*M), **3e** (12.5 *μ*M), **3f** (12.5 to 50 *μ*M), and **4b** (12.5 *μ*M). PZQ (10 *μ*M) caused 100% of death of the parasites without separation of worms. 

**Figure 1:**
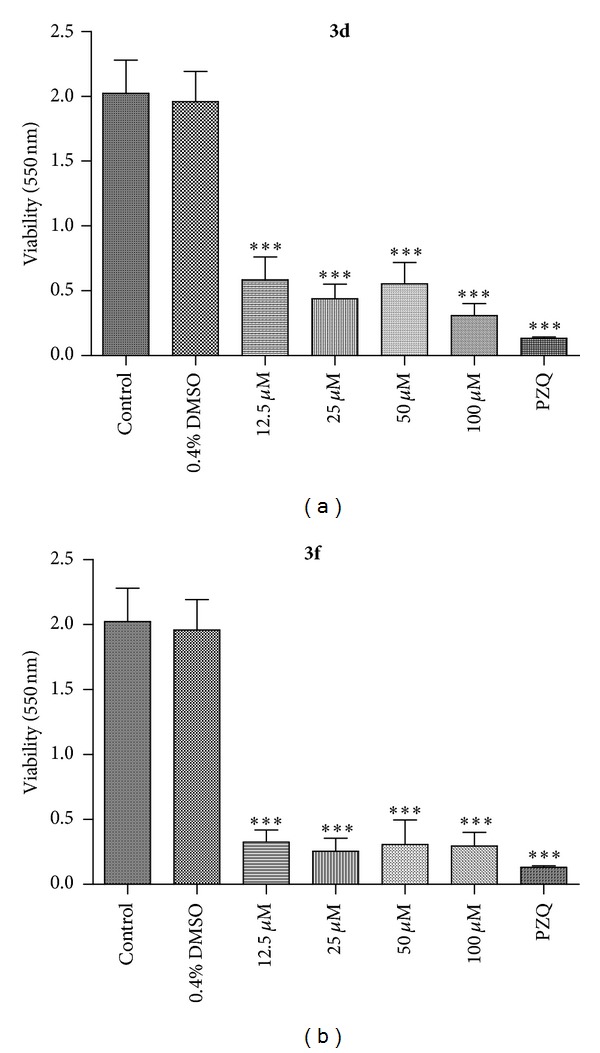
*In vitro *effects of active diamines **3d** and **3f** on the viability of the *S. mansoni* adult worms. Pairs of adult worms were treated with samples in different concentrations for 72 h, and the viability was measured by using the MTT assay at 550 nm. RPMI 1640 medium and 0.4% DMSO in RPMI 1640 medium were used as negative controls. Praziquantel (PZQ, 10 *μ*M) was used as positive control group. The viability was expressed as mean of the absorbance values from four experiments. ***P < 0.001.

After preliminary screenings and mortality analysis, the viability and egg development assays of adult *S. mansoni* worms were evaluated by incubation with different concentrations of the most active compounds **3d** and **3f** (12.5 to 100 *μ*M) (Figure 1). In the groups treated with **3f** (25 to 100 *μ*M), the viability of adult worms was statistically similar to PZQ (10 *μ*M) after 72 hours of incubation. Similarly, groups of adult worms treated with **3d** (100 to 100 *μ*M) displayed reduced viability compared to the positive control PZQ. It is important to point out that compounds **3d** and **3f** showed high activity in this assay, but a dose-response effect was not observed in comparison with the negative control group, treated with RPMI 1640 medium, in which the worms remained viable during 72 h of incubation (Figure 1).

**Figure 2:**
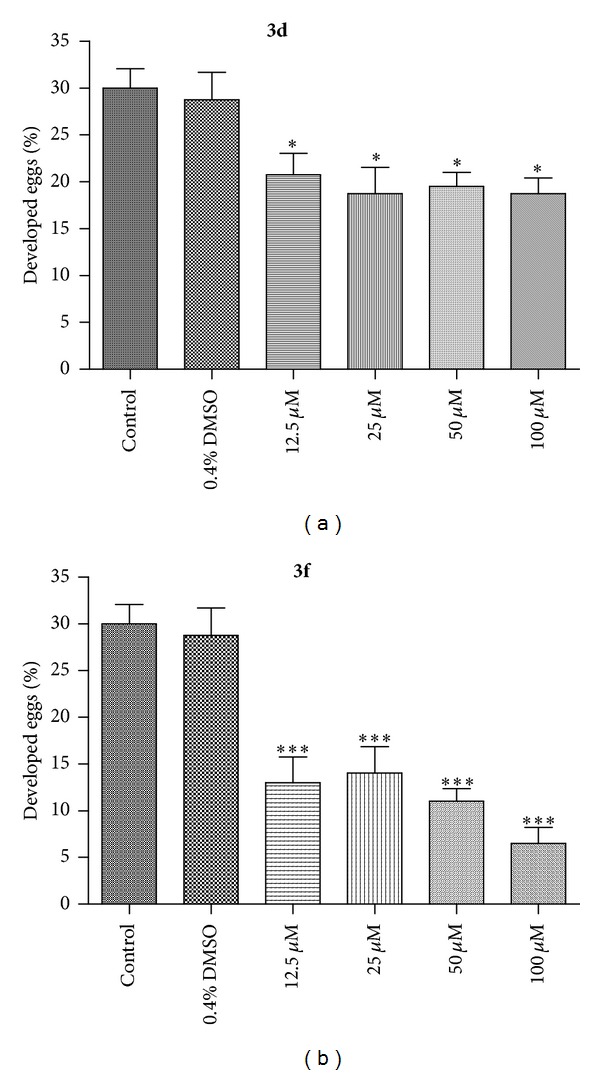
*In vitro *active diamines **3d** and **3f** on egg development (quantitative analysis of the development phenotype). After treatment, the eggs were microscopically examined and scored as developed or undeveloped based on the presence or absence of the miracidium. Data are presented as the mean of developed eggs from four separate experiments. *P < 0.05, ***P < 0.001.

In order to evaluate the percentage of developed eggs produced by adult worms of *S. mansoni*, groups of parasites were incubated with **3d** and **3f** (12.5 to 100 *μ*M) and monitored for 72 hours. As shown in Figure 2, compounds **3d** (12.5 to 100 *μ*M) and **3f** (12.5 to 100 *μ*M) showed significant decrease in the production of developed eggs when compared with the negative control group but not in a dose-response dependent manner. Despite the difference in groups, this *in vitro* bioassay showed a significant reduction in the mean egg development output after exposure to concentrations of the active compounds **3d** and **3f**. According to the literature, 20–30% of the eggs produced by adult worms in the first two days of *in vitro* culture went through the six development stages in five days until the formation of the miracidium inside could be considered as fully developed [5, 8, 24]. Also, the presence of *S. mansoni* eggs in the host tissues has been reported to be closely related to the pathology of human schistosomiasis, and egg production is responsible for the transmission of the parasite schistosome and the maintenance of its life cycle [8, 27]. In the present study, we observed that compounds **3d** and **3f** reduced egg development output by affecting pairing or mortality of female worms. However, it is still unknown whether these antifecundity effects of compounds **3d** and **3f** were the result of more specific inhibition of reproductive processes.

Considering the most active compounds, the mechanism by which lipophilic diamines **3d** and **3f** exert their *in vitro* schistosomicidal effect is not clear. One of the possible targets for the diamines **3d** and **3f** actions is the damage of schistosome metabolism. It has been reported that polyamines are essential for cell proliferation and differentiation of some parasites. It has been shown that interfering with their function or biosynthesis, polyamines can block cellular growth [10, 11]. Also, it was suggested that diamines containing long alkyl chains, such as compound **3f**, seem to impair the parasite biosynthetic metabolism, leading to parasite death [10–17]. However, taken all results together, even **3d** and **3f** have similar chemical structures; they may have different mechanisms involved in the parasite's death, since they affect in a different way male and female adult schistosomes. Due to the complexity of drug mechanisms and their mode of action, future biological experiments are necessary to clarify their mechanisms of action.

## 4. Conclusion

Diamines and amino alcohols were prepared by synthetic methods and submitted to *in vitro* evaluation against adult worms of *Schistosoma mansoni*. The present results indicate that alkylated diamines and amino alcohols are potentially antiparasitic compounds that may be useful starting points to find an ideal lead for the development of new schistosomicidal agents. We have identified some promising compounds that demonstrate high *in vitro *activity against adult schistosome, especially to male worms. However, further *in vitro *and *in vivo *studies are necessary to fully determine the potential chemotherapeutic efficacy of these compounds in the schistosome treatment.

## Figures and Tables

**Table 1:** *In vitro* effects of alkylated diamines and amino alcohols against adult worms of *S. mansoni*.

Samples^b^	% worm separation^e^	% worm mortality^e^	Significant reduction in motor activity (%)^e^	log⁡(*P*)
Control^a^	0.0	0.0	0.0	
0.4% DMSO	0.0	0.0	0.0	
PZQ^c^	0.0	100	0.0	
Compounds^d^				
** 3a**	0.0	0.0	0.0	1.15
** 3b**	50	100	0.0	1.99
** 3c**	0.0	0.0	0.0	2.82
** 3d**	100	100	50	3.65
** 3e**	75	100	0.0	4.49
** 3f**	100	100	50	3.55
** 3g**	75	0.0	100	8.65
** 3h**	25	100	25	4.38
** 4a**	0.0	100	25	4.62
** 4b**	50	100	50	5.30
** 4c**	0.0	0.0	75	4.76
** 4d**	0.0	100	25	4.87
** 10a**	0.0	100	50	2.55
** 10b**	0.0	100	50	3.39
** 10c**	0.0	0.0	0.0	4.22
** 10d**	0.0	100	50	5.06

^a^RPMI 1640; ^b^period of incubation: 72 h; ^c^tested at concentration of 10 *μ*M; ^d^tested at concentration of 100 *μ*M; ^e^percentages relative to the 8 adult worm pairs investigated. Slight was defined as a reduction in movement compared with the negative control. Significant was defined as minimal movement observed for 1 minute. Dead worm was defined when no movement was observed for at least 2 minutes of examination. log⁡(*P*) values were calculated using Chemdraw Ultra 12 software (trial version).

**Table 2:** *In vitro* effects of active diamines and amino alcohols against adult worms of *S. mansoni*.

Samples^b^	% worm separation^d^	% worm mortality^d^		Significant reduction in motor activity (%)^d^
		M	F	
Control^a^	0.0	0.0	0.0	0.0
0.4% DMSO	0.0	0.0	0.0	0.0
PZQ^c^	0.0	100	100	100
**3b**				
12.5 *μ*M	0.0	0.0	0.0	0.0
25 *μ*M	0.0	0.0	0.0	0.0
50 *μ*M	0.0	0.0	0.0	0.0
**3d**				
12.5 *μ*M	50	100	100	0.0
25 *μ*M	50	100	100	0.0
50 *μ*M	50	100	100	0.0
**3e**				
12.5 *μ*M	50	0.0	0.0	0.0
25 *μ*M	100	0.0	0.0	0.0
50 *μ*M	100	0.0	0.0	0.0
**3f**				
12.5 *μ*M	50	100	0.0	0.0
25 *μ*M	50	100	0.0	0.0
50 *μ*M	50	100	0.0	0.0
**3h**				
12.5 *μ*M	0.0	0.0	0.0	0.0
25 *μ*M	0.0	0.0	0.0	0.0
50 *μ*M	0.0	0.0	0.0	0.0
**4a**				
12.5 *μ*M	0.0	0.0	0.0	0.0
25 *μ*M	0.0	0.0	0.0	0.0
50 *μ*M	0.0	75	25	0.0
**4b**				
12.5 *μ*M	50	0.0	0.0	0.0
25 *μ*M	100	0.0	0.0	0.0
50 *μ*M	100	0.0	0.0	0.0
**4d**				
12.5 *μ*M	0.0	0.0	0.0	0.0
25 *μ*M	0.0	0.0	0.0	0.0
50 *μ*M	0.0	0.0	0.0	0.0
**10a**				
12.5 *μ*M	0.0	0.0	0.0	0.0
25 *μ*M	0.0	0.0	0.0	0.0
50 *μ*M	0.0	75	25	0.0
**10b**				
12.5 *μ*M	0.0	0.0	0.0	0.0
25 *μ*M	0.0	100	0.0	0.0
50 *μ*M	0.0	100	25	0.0
**10d**				
12.5 *μ*M	0.0	0.0	0.0	0.0
25 *μ*M	0.0	100	25	0.0
50 *μ*M	0.0	100	50	0.0

^a^RPMI 1640; ^b^period of incubation: 72 h; ^c^tested at 10 *μ*M; ^d^percentages relative to the 8 adult worm pairs investigated. M: males; F: females. Slight was defined as a reduction in movement compared with the negative control. Significant was defined as minimal movement observed for 1 minute. Dead worm was defined when no movement was observed for at least 2 minutes of examination.
